# Sensitivity of air quality to vehicle ammonia emissions in the United States

**DOI:** 10.1016/j.atmosenv.2024.120484

**Published:** 2024-06-15

**Authors:** Claudia Toro, Darrell Sonntag, Jesse Bash, Guy Burke, Benjamin N. Murphy, Karl M. Seltzer, Heather Simon, Mark W. Shephard, Karen E. Cady-Pereira

**Affiliations:** a US Environmental Protection Agency, Office of Transportation and Air Quality, Ann Arbor, MI, USA; b Department of Civil and Construction Engineering, Brigham Young University, Provo, UT, USA; c US Environmental Protection Agency, Office of Research and Development, RTP, NC, USA; d US Environmental Protection Agency, Region 2, New York, NY, USA; e US Environmental Protection Agency, Office of Air Quality Planning and Standards, RTP, NC, USA; f Environment and Climate Change Canada, Toronto, ON, Canada; g Atmospheric and Environmental Research, Lexington, MA, USA

**Keywords:** Ammonia, Vehicles, Particulate matter, Mobile sources, Urban emissions

## Abstract

The US Environmental Protection Agency (EPA) estimates on-road vehicles emissions using the Motor Vehicle Emission Simulator (MOVES). We developed updated ammonia emission rates for MOVES based on road-side exhaust emission measurements of light-duty gasoline and heavy-duty diesel vehicles. The resulting nationwide on-road vehicle ammonia emissions are 1.8, 2.1, 1.8, and 1.6 times higher than the MOVES3 estimates for calendar years 2010, 2017, 2024, and 2035, respectively, primarily due to an increase in light-duty gasoline vehicle NH_3_ emission rates. We conducted an air quality simulation using the Community Multi-Scale Air Quality (CMAQv5.3.2) model to evaluate the sensitivity of modeled ammonia and fine particulate matter (PM_2.5_) concentrations in calendar year 2017 using the updated on-road vehicle ammonia emissions. The average monthly urban ammonia ambient concentrations increased by up to 2.3 ppb_v_ in January and 3.0 ppb_v_ in July. The updated on-road NH_3_ emission rates resulted in better agreement of modeled ammonia concentrations with 2017 annual average ambient ammonia measurements, reducing model bias by 5.8 % in the Northeast region. Modeled average winter PM_2.5_ concentrations increased in urban areas, including enhancements of up to 0.5 μg/m^3^ in the northeast United States. The updated ammonia emission rates have been incorporated in MOVES4 and will be used in future versions of the NEI and EPA’s modeling platforms.

## Graphical Abstract

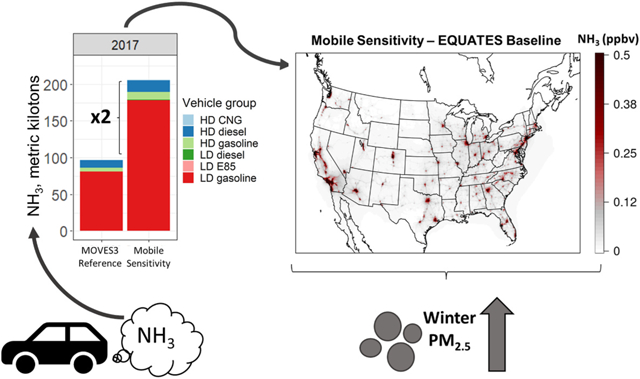

## 1. Introduction

Atmospheric ammonia (NH_3_) concentrations have increased by more than 40% in the United States from 2008 to 2018 based on both ground-level (Yao and Zhang, 2019) and satellite measurements (Damme et al., 2021). The US EPA has reported an increase in NH_3_ emissions of 14% in the US in the same time period, and a subsequent increase in NH_3_ emissions of 2% between 2018 and 2022. (US EPA) Some studies have reported that lower sulfur dioxide (SO_2_) and nitrogen oxides (NOx) emissions result in lower formation of ammonium sulfate and ammonium nitrate, and a subsequent increase in ambient gas-phase NH_3_, (Saylor et al., 2015; Schiferl et al., 2016; Yao and Zhang, 2019) suggesting that controlling NH_3_ emissions would also reduce ambient fine particulate matter (PM_2.5_) (Arter et al., 2021; Damme et al., 2021; Paulot and Jacob, 2014).

National-scale NH_3_ emissions are dominated by agriculture, contributing over 80% of emissions in the 2017 and 2020 National Emission Inventory (NEI) (US EPA, 2021; US EPA, 2023). However, in urban areas, on-road vehicles are important sources of NH_3_ emissions (Cao et al., 2022; Chen et al., 2022; Sun et al., 2017) which are released as an unintended byproduct of aftertreatment systems. In gasoline vehicles, NH_3_ is formed from the catalytic reduction of nitrogen oxide (NO) across the three-way catalyst during fuel rich conditions (Easter and Bohac, 2016). Conventional diesel vehicles (i.e., model years prior to 2010) have rather low NH_3_ emissions. To comply with US heavy-duty diesel 2010 model year emission standards (US EPAb), modern heavy-duty diesel vehicles are equipped with selective catalytic reduction systems to control nitrogen oxide (NO_X_) emissions, which actively inject urea into the aftertreatment system. The urea decomposes into NH_3_ in the aftertreatment system (Jeon et al., 2016), and any unreacted NH_3_ escapes the aftertreatment system leading to NH_3_ emissions (Khalek et al., 2015). Several recent studies have suggested that combustion-related NH_3_ emissions, including on-road vehicle emissions, are underestimated in emission inventories (Cao et al., 2022; Chen et al., 2022; Emery et al., 2020; Farren et al., 2020; Moravek et al., 2019; Sun et al., 2017). The underestimation of NH_3_ in vehicle emissions could lead to underestimation of their contribution to ambient particulate matter and nitrogen deposition pollution.

On-road vehicle emissions for the NEI are estimated using the Motor Vehicle Emission Simulator (MOVES) (US EPA Motor Vehicle Emission Simulator (MOVES)) for all States except California, which are generally estimated using the EMission FACtor (EMFAC) (CARB, 2021) model. MOVES contains a database of on-road running exhaust vehicle emission rates as a function of vehicle class, fuel type, operating mode, model year, and vehicle age in units of mass per time (gram/hour). MOVES uses emission rates coupled with estimates of vehicle activity to estimate vehicle emissions by county for all calendar years between 1999 and 2060 (US EPA, 2021). MOVES simulations used for the NEI account for county-level differences in vehicle fleet composition (vehicle classes), vehicle age distributions, and inputs that impact operating mode distributions (e.g., vehicle speeds and roadway type distributions).

MOVES3 (and earlier versions) calculates vehicle NH_3_ emission rates based on data from a study carried out in the early 2000s (Durbin et al., 2002; US EPA, 2010). These data has been the basis for national NH_3_ emissions developed for NEIs up to its most recent version (2020NEI) (US EPA, 2015; US EPA, 2018; US EPA, 2021; US EPA, 2023). EMFAC versions preceding EMFAC2021 did not include NH_3_ vehicle emission rates, and MOVES3 was used to generate California emissions included in all NEI versions mentioned previously as well as in EPA’s Air QUality TimE Series (EQUATES) project (Foley et al., 2023). The latest version of the MOVES model, MOVES4, (US EPA, 2023a) incorporates updated NH_3_ emission rates for on-road vehicles based on roadside remote sensing measurements. The new rates are a better representation of the current US fleet and increase significantly on-road NH_3_ emissions. In this study, we evaluated the modeled air quality impacts of using the 2017 EQUATES dataset (which uses the 2017 NEI as base year) with adjusted NH_3_ on-road vehicle emissions based on measurements from roadside emission studies, following a similar methodology to that used in the development of emission rates for MOVES4. Our goal is to quantify the effect of using on-road NH_3_ emission rates, based on recent real-world measurements, on the simulation of NH_3_ and PM_2.5_ levels in urban areas.

## 2. Methods

### 2.1. On-road vehicle ammonia emission rates

We revised the NH_3_ emission rates in MOVES3 for on-road vehicles using data from roadside measurement studies (Preble et al., 2019; Fuel Efficiency Automobile Test Data) from both light-duty gasoline and heavy-duty diesel vehicles. The NH_3_ emission rates in MOVES3 and earlier versions were based on a study with a limited number of vehicles, of model year pre-2000, sampled in laboratory conditions (Durbin et al., 2002; US EPA, 2010). By using road-side measurements, the updated NH_3_ emission rates in this study are based on hundreds to thousands of in-use vehicles, including high-emitting vehicles that contribute disproportionately to the emissions inventory. The methodology to develop ammonia emission rates for light-duty and heavy-duty vehicles using roadside measurements is described in detail in the MOVES4 technical documentation (US EPA, 2023b; US EPA, 2023c). However, we provide a brief overview of the dataset and general approach used below. We further note that the work described in this paper was developed using a first draft of the emission rates developed for MOVES4. Nonetheless, as discussed in Section S2, the changes to the ammonia emission rates incorporated into the final MOVES4 have a minimal impact in the adjustments developed for this work.

For light-duty gasoline vehicles, we analyzed fuel-based NH_3_ emissions measured by researchers at the University of Denver using a roadside remote sensing device called the Fuel Efficiency Automobile Test (FEAT) (Fuel Efficiency Automobile Test Data). The emissions data collected by the University of Denver using FEAT are publicly available and contains over 335,000 light-duty gasoline vehicle-specific NH_3_ observations collected at seven different locations across the United States from 2005 to 2020 (see Tables S–1). Fuel-specific light-duty gasoline vehicle NH_3_ rates derived from FEAT compare well across different locations in the US (Tables S–1), and to on-road and roadway tunnel NH_3_ measurements made by other researchers at different locations in the United States, Europe, and Beijing China (Sun et al., 2017). Using FEAT-reported measurements, we developed average fuel-based NH_3_ emission rates for light-duty vehicles by vehicle class (light-duty car or light-duty truck), model year and age (US EPA, 2023b).

For heavy-duty diesel vehicles, we utilized NH_3_ emission rates from a study by Preble et al. (Preble et al., 2019), who sampled exhaust plumes of over 900 individual heavy-duty vehicles at the Caldecott Tunnel near Oakland, California in 2018 (see Section S1.2). By matching license plate images to state truck registration databases, they associated the measurements with vehicle information including engine model year and type of aftertreatment system. Preble et al. reported average emission rates for heavy-duty diesel vehicles by model year ranges and type of exhaust aftertreatment system, including use of diesel particulate filters and selective catalytic reduction. This is an advantage from the perspective of MOVES modeling as we can assign emission rates to specific heavy-duty vehicle populations. The fleet-average fuel-based NH_3_ emission rates reported by Preble et al. compare well to other heavy-duty diesel fleet-averages as discussed in Section S1.2 (Tables S–3) and references therein.

Using the fuel-based NH_3_ emission rates from the road-side studies, we estimated the updated MOVES NH_3_ emission rates (in units of g/hour, classified by operating mode) for light-duty gasoline and heavy-duty diesel vehicles. The conversion from fuel-based rates (g NH_3_/kg-fuel) to mass rates (g NH_3_/hr) involves multiplying the fuel-based observations for a specific vehicle type-model year group by the corresponding MOVES fuel consumption rate (kg-fuel/hr) as described in Section S1.3. A limitation of using remote sensing data for our purposes is that the measurements are taken under a narrow range of driving conditions, generally low speed and acceleration, limiting the characterization of vehicle emissions across the range of operating modes. This is important for emissions that correlate with vehicle specific power, as expected for ammonia. We can account for this in our methodology because the fuel consumption rates used in the conversion to mass rates are defined across the range of operating modes represented in MOVES. Thus, the resulting ammonia mass rates will have the same relative increase with vehicle specific power than the corresponding fuel consumption rates. However, it is possible that the mass rates derived might still underestimate emission rates at high acceleration conditions. This uncertainty can only be minimized by using measurements that capture a wider range of operating conditions (e.g., dynamometer measurements, Portable Emissions Measurement Systems (PEMS)), but these data were not available at the time of developing this work. Therefore, validating the emission rates derived here against data representing the full range of operating conditions will be the focus of future analyses.

**Fig. 1.**
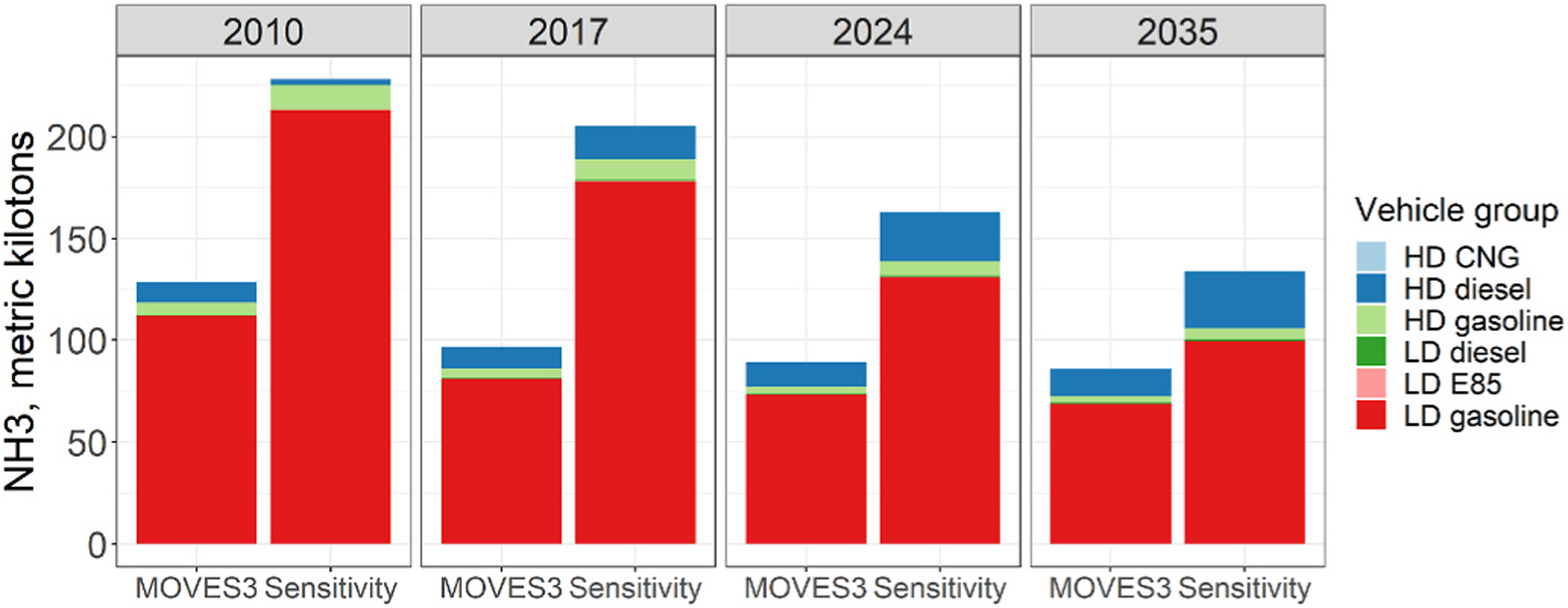
Annual national on-road vehicle NH_3_ emissions estimated from MOVES3 and Sensitivity cases by calendar year and vehicle group. The vehicle groups are a combination of vehicle type and fuel type. HD = heavy-duty; LD = light-duty. CNG = compressed natural gas; E85 = ethanol-gasoline blend with ~85% ethanol. Note that the modeling uses default MOVES3 national activity in both cases.

After deriving mass rates, we estimated national on-road vehicle NH_3_ emissions inventories for four calendar years, using the default MOVES3 emission rates (MOVES3 reference case) and the updated NH_3_ emission rates, referred to as Sensitivity case (see Section S2). Fig. 1 compares the national NH_3_ emissions between MOVES3 and the Sensitivity cases by vehicle type and fuel type for four calendar years. Light-duty gasoline vehicles contribute between 79% and 87% of on-road vehicle NH_3_ emissions in MOVES3, with a similar range (74%–93%) for the Sensitivity case. Overall, the modeled emissions indicate a decreasing trend in NH_3_ with calendar year; this is largely explained by the fleet turnover of LD gasoline vehicles (Section S4), which are the major contributors to the NH_3_ inventory.

The on-road vehicle NH_3_ emissions estimated in the sensitivity case were higher than MOVES3 for all four years evaluated. The largest increase in NH_3_ emissions in the sensitivity case occurs in calendar years 2010, 2017, and 2024 due to the substantial increase in NH_3_ emission rates for the model year 2000–2016 light-duty gasoline vehicles (Figure S-3). Heavy-duty diesel vehicles make up an increasing share of NH_3_ emissions in future years due to the penetration of model year 2010 and later heavy-duty diesel vehicles. These vehicles use selective catalytic reduction aftertreatment systems, which have higher NH_3_ emission rates (g/km) than comparable model year gasoline vehicles (Figure S-4). Additional details on the MOVES simulations are discussed in the supporting information.

For evaluating the impact of these increased on-road NH_3_ emissions on ambient air pollution concentrations using photochemical models, we developed average nationwide, calendar year (CY) specific on-road vehicle NH_3_emissions scaling factors (SF) using Equation (1):

Equation 1 $$NH_{3}SF_{CY}=\left(\frac{\text{MOVES}3_{NH_{3}\text{ emissions, }CY,\text{ Sensitivity }}}{\text{MOVES}3_{NH_{3}\text{ emissions, }CY,\text{ Baseline }}}\right)$$

Calculations using Equation (1) were performed for the vehicle groups presented in Fig. 1 and then grouped into on-road diesel and non-diesel SF for purposes of the air quality sensitivity simulation (see Section S3). National on-road NH_3_ emissions for the sensitivity cases were 1.8, 2.1, 1.8, and 1.6 times higher than those developed using MOVES3 in calendar years 2010, 2017, 2024, and 2035, respectively (Tables S–4). These factors agree with the low end of the range of NH_3_ underestimation presented by studies suggesting low NH_3_ onroad inventories using different methodologies. In particular, Sun et al. measured on-road NH_3_: CO_2_ ratios and estimated that the US national on-road NH_3_ inventory for 2011 was at least a factor of 2 low (Sun et al., 2017); Cao et al. suggested that vehicle NH_3_ emissions in the US were underestimated by a factor ranging between 1.8 and 4.9 using satellite observations and fuel-based inventories (Cao et al., 2022). Fenn et al. (2018) used on-road measurements to estimate that vehicular NH_3_ emissions are 2.9 times greater than those estimated in the 2011 NEI; Walters et al. (2022) did not propose a factor, but suggested that the source characterization of NH_3_ in the 2014 NEI might be underestimating vehicular contribution and overestimating residential combustion sources for a location in the northeast US. For the purposes of our air quality simulation, the NH_3_ SF developed using Equation (1) were 2.1 for on-road non-diesel and 1.5 for on-road diesel sources (Section S3).

### 2.2. Air quality model simulations

The impact of increased on-road NH_3_ emissions on ambient air quality was estimated using a CMAQ v5.3.2 annual 2017 Ammonia Mobile Emissions Sensitivity simulation, hereafter referred to as AMES. We selected the calendar year 2017 for our air quality simulation to leverage previous work done by Benish et al. (2022) which is based on EPA’s EQUATES project and incorporates the most up to date understanding of simulated deposition trends in the US, thus serving as base case for our sensitivity analysis. Mobile on-road NH_3_ emission rates were adjusted using the Detailed Emissions Scaling, Isolation and Diagnostic (DESID) (Murphy et al., 2021) tool available in CMAQ v5.3.2. The DESID tool allows for the adjustment of emissions based on the emission sector and/or geographic region. We represent the updates to on-road NH_3_ emissions by applying the fuel-specific SF developed from national estimates for the year 2017 to the onroad sector, as described previously. The on-road diesel and on-road non-diesel mobile emission rates for NH_3_ for 2017 were increased by 1.5 and 2.1, respectively (Section S3) for the conterminous US portion of the domain. With the exception of NH_3_ emission factors for on-road mobile sources, this simulation was identical to the EQUATES model simulation for 2017 described in Benish et al. (2022), which serves as the base case. Annual 2017 CMAQv5.3.2 simulations were completed for the contiguous U.S. domain using 12 km horizontal grid spacing and 35 vertical layers. Anthropogenic emission inputs were based on the 2017 National Emission Inventory (NEI) (US EPA, 2021) and biogenic emissions were run inline using the Biogenic Emission Inventory System (BEIS) (Bash et al., 2016), following methods used in the EQUATES simulations. Meteorological inputs were generated from the Weather Research Forecasting (WRF) model version 4.1.1 and lateral boundary conditions were provided by the EQUATES simulations. The Surface Tiled Aerosol and Gaseous Deposition (STAGE) option in CMAQ v5.3.2 was used to estimate atmospheric dry deposition rates utilizing the bidirectional exchange option for NH_3_ from natural and agricultural land uses (Appel et al., 2021; Galmarini et al., 2021). Annual 2017 CMAQ v5.3.2 modeled results were evaluated against Cross Infrared Sounder (CrIS) satellite (Shephard et al., 2020), Ammonia Monitoring Network (AMoN) surface NH_3_ observations, the U.S. EPA’s Air Quality System (AQS) ambient PM_2.5_, Chemical Speciation Network (CSN) ambient nitrate $\left({\text{NO}}_{3}^{-}\right)$ PM_2.5_, Interagency of PROtected Visual Environments (IMPROVE) monitoring network ambient ${\text{NO}}_{3}^{-}$ PM_2.5_ and the National Atmospheric Deposition Program (NADP) National Trends Network (NTN) for wet deposition of ammonium $\left({\text{NH}}_{4}^{+}\right)$ observations. Estimated model ambient concentrations and deposition totals were paired in space and time with these observations using the Atmospheric Model Evaluation Tool (Appel et al., 2011) version 1.5.

## 3. Results and discussion

### 3.1. Air quality sensitivity - including updated ammonia and particulate matter concentrations

**Fig. 2.**
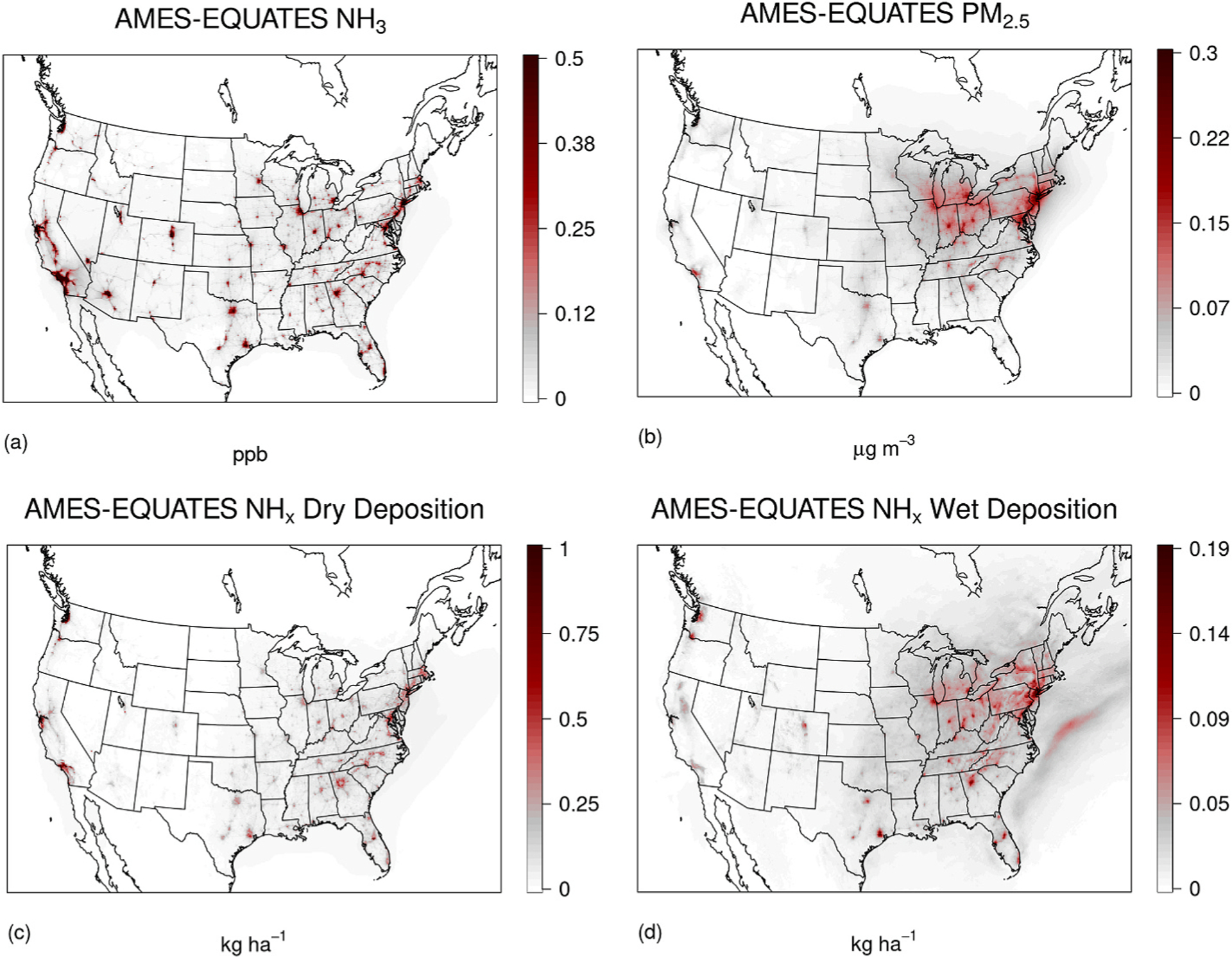
Annual model differences (AMES – EQUATES) in surface layer NH_3_ concentrations in ppb_v_ (a), PM_2.5_ in μg m^−3^ (b), NH_x_, NH_3_ + Aerosol ${\text{NH}}_{4}^{+}$ dry deposition in kg ha^−1^ (c), NH_x_ wet deposition in kg ha^−1^ (d).

**Fig. 3.**
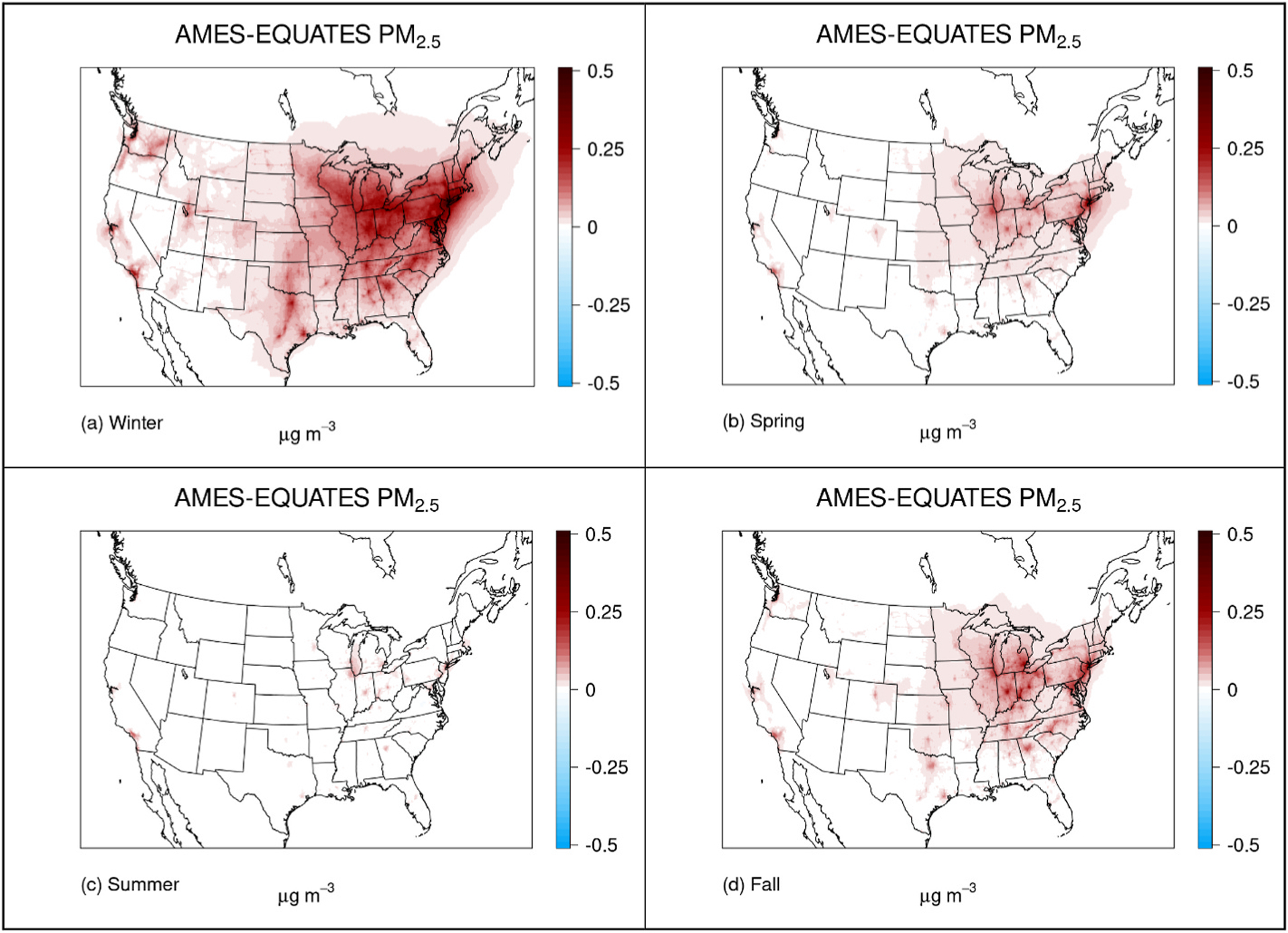
Seasonal model differences (AMES – EQUATES) in surface layer PM_2.5_ concentrations μg m^−3^ 3 December, January, and February (a), March, April, and May (b), June, July, and August (c), September, October, and December (d).

The AMES case resulted in an increase in modeled ambient NH_3_ concentrations in the model domain, primarily in urban areas and transportation corridors (Fig. 2a, Figure S-6a). These increases were relatively consistent over the year with a maximum increase in monthly mean ambient NH_3_ concentration of 2.3 ppb_v_ in January and 3.0 ppb_v_ in July (Figure S-11) in Southern California around Los Angeles, likely due to the combination of high traffic emissions and higher temperatures. The increase in NH_3_ emissions resulted in increases in modeled PM_2.5_ concentrations and in wet and dry deposition in urban regions (Fig. 2b–d, Figure S-6b–d). The modeled increased aerosol load was predominantly composed of NH_4_NO_3_ in agreement with the findings of Kim et al. (2023), which is modeled in thermodynamic equilibrium with ambient NH_3_ and HNO_3_ (Meng and Seinfeld, 1996). Therefore, PM_2.5_ increases were largely limited to the cooler months when conditions favored NH_4_NO_3_ formation (see Table 1 and Fig. 3). Increases in atmospheric NH_3_ were proportionally larger than the increase in the atmospheric aerosol burden (Figure S-6) due to the conditions favoring NH_4_NO_3_ formation and dry and wet deposition losses of NH_3_. On a population-weighted basis, which refers to the average exposure of the national population, annual average PM_2.5_ concentrations increased most in the greater New York City region (0.2–0.3 μg m^−3^), with wintertime increases approximately 50% higher. Modeling studies have indicated that aerosol formation in the Northeastern US is NH_3_ limited (Pye et al., 2009) in agreement with the simulations presented here. In southern California, where ambient ammonia concentrations featured the largest increases, annual average PM_2.5_ concentrations increased 0.1 μg m^−3^.

Recent ambient observations have indicated approximately two-fold increase in total nitrogen deposition in urban areas largely composed of ammonia and ammonium relative to their corresponding upwind rural background (Decina et al., 2020). This sensitivity increased modeled NH_x_ (gaseous NH_3_ + aerosol ${\text{NH}}_{4}^{+}$) deposition in urban areas (areas with higher levels of onroad emissions) up to 1.3 kg N ha^−1^ year^−1^ resulting in a mean 15% and 9% increase in modeled NH_x_ and total N deposition in urban areas, respectively. The maximum deposition increase is approximately equivalent to the threshold at which lichen communities display adverse ecological effects, (Geiser et al., 2021) known as the ecosystems critical load, and approximately 20%–25% of the critical load for sensitive tree species (Pavlovic et al., 2023).

### 3.2. Evaluation against CrIS satellite and ground-based network observation

The contribution of the AMES case to the modeled mean annual NH_3_ urban concentrations were as large as 144% and with a mean increase of approximately 17% for urban areas over the model domain (Fig. 2). We compared our results to observations from the Cross-track Infrared Sounder (CrIS) satellite which provides global observations of ambient NH_3_ concentrations twice a day (1330 and 0130 local time). The annual 2017 CMAQ AMES case compared well against CrIS satellite NH_3_ observations (Shephard et al., 2020) and broadly captured both magnitude and spatial variability of the observations (Figure S-7). Mobile NH_3_ emissions differences between AMES and EQUATES were small compared to agricultural sector NH_3_ emissions in the modeling domain on a national level, leading to relatively small model differences in NH_3_ and PM_2.5_ concentrations between the AMES and EQUATES cases during the midday CrIS overpass. Thus, evaluation against CrIS observations largely illustrates the general ability to capture the large spatial features and magnitudes of the observed concentration fields by the CMAQ modeled NH_3_ concentration fields. The AMES simulation had a larger impact when comparing to surface network NH_3_, PM_2.5_ and wet deposition observations because these networks sites are typically not located in heavily agricultural areas (Table 1). (Cao et al., 2022) Modeled NH_3_ concentrations in the AMES case were generally improved when evaluated against AMoN surface observations in the contiguous United States, with a model bias reduction of 5.8% in the Northeast and 2.3% for all observations. The AMES case also resulted in small improvements in model predictions of NH_4+_ wet deposition when compared to observations at National Atmospheric Deposition Program (NADP) National Trends Network (NTN) monitoring sites. The small differences in model evaluations for the NADP and AMoN networks are likely due to the underrepresentation of urban areas in monitoring networks that were initially designed to represent more regional patterns (Bettez and Groffman, 2013). Over the contiguous U.S., PM_2.5_ and ${\text{NO}}_{3}^{-}$ PM_2.5_ model biases were lower in the AMES simulation at AQS monitoring sites, though the improvements were not uniform (Table 1). For example, there were increased PM_2.5_ biases at times and locations where PM_2.5_, and specifically the ${\text{NO}}_{3}^{-}$ component, were already overestimated in the EQUATES simulation (Figures S-8 to S-10). This is most clear during the winter months where ${\text{NO}}_{3}^{-}$ formation is more likely due to lower temperatures. The location of monitoring network sites impacted the model evaluation against observed values. For example, CSN aerosol observations are largely located in more urban areas while IMPROVE observations are located in more rural sites and the impact of the AMES sensitivity on the model evaluation are largest at CSN sites reflecting the larger contribution of mobile emissions in urban areas (Table 1).

## 4. Conclusions

This study explores the sensitivity of modeled PM_2.5_ to the increase in onroad ammonia emissions resulting from updating ammonia emission rates for light and heavy-duty vehicles in MOVES, using on-road remote sensing observations. The modeled PM_2.5_ increases resulting from updated on-road NH_3_ emission rates in the 2017 sensitivity case result in increases of up to 0.3 μg m^−3^ (5%) in modeled annual average PM_2.5_ concentrations. Although our study is based on national scale factors that might differ depending on the local fleet, the highest PM_2.5_ values were modeled as occurring in the greater New York City region (0.2–0.3 μg m^−3^), with wintertime increases approximately 50% higher. In Southern California, where modeled ambient ammonia concentrations featured the largest increases, the model predicted an increase of 0.1 μg m^−3^ in annual average PM_2.5_ concentrations. The AMES case presented here shows moderate improvements in the bias between modeled NH_3_ concentrations and AMoN surface observations, particularly in urban areas in the Northeastern region, and to a smaller degree for modeled winter and spring PM_2.5_ and ${\text{NH}}_{4}^{+}$ wet deposition across the country. Many of the areas that exhibited the largest changes in the model simulations, particularly in ambient NH_3_ and wet deposition, were not well represented by network observations and highlight the need for additional monitoring of PM_2.5_ composition and NH_3_. The siting of PM_2.5_ observations has an impact on the model evaluation, Table 1, with a larger impact observed at urban CSN than rural IMPROVE sites. This highlights the need for additional network observations of PM_2.5_ and particulate matter precursors. Despite the paucity of urban monitoring for NH_3_ and wet deposition, the updates to NH_3_ emission rates in MOVES using this bottom-up emissions approach are supported by the improvement in air quality modeling estimates when evaluated against network observations. Since on-road mobile NH_3_ is emitted primarily during running operation, it is possible to leverage the roadside measurements available for thousands of vehicles for this purpose, albeit limited to low speed and acceleration conditions. This limitation can potentially result in an underestimation of the scaling factors derived here, and highlights the need of emissions measurements across the range of operating conditions. While our air quality analysis focused on 2017, we expect that the impact of updated onroad NH_3_ emissions will decrease in future years as the vehicular fleet evolves, removing older light-duty vehicles which are the major contributors to the urban NH_3_ inventory. The results presented in this work provide an insight into the impact of new urban ammonia inventories developed with MOVES4 and future versions of the NEI on modeling of PM_2.5_.

## Supplementary Material

Toro_etal_NH3mobile_2024_SI

## Figures and Tables

**Table 1:** Modeled 2017 normalized annual mean biases (%) of the Ammonia Mobile Emissions Sensitivity (AMES) and EQUATES simulations compared against air quality monitoring network observations for the contiguous United States for December, January, and February (DJF), March, April, and May (MAM), June, July, and August (JJA), and September, October, and November (SON). Negative values indicate an underestimate and positive values indicate an overestimate of the observed values by the AMES simulation.

Season	AMoN NH_3_		CSN PM_2.5_ ${NO}_{3}^{-}$		IMPROVE PM_2.5_ ${NO}_{3}^{-}$		AQS PM_2.5_		NADP ${NH}_{4}^{+}$ Wet Deposition	
	AMES	EQUATES	AMES	EQUATES	AMES	EQUATES	AMES	EQUATES	AMES	EQUATES
**Winter (DJF)**	−46.3	−50.0	1.8	−5	1.4	−6.2	−11.0	−12.6	−52.9	−55.5
**Spring (MAM)**	−43.3	−45.1	−3	−11.6	−21	−25.7	−1.8	−3.0	−41.8	−42.7
**Summer (JJA)**	−8.9	−10.7	−17.9	−24	−39.2	−40.9	−18.0	−18.3	−11.7	−15.0
**Fall (SON)**	−15.5	−18.9	27	14.6	−11.3	−17.7	−8.4	−9.4	−37.5	−39.1

## Data Availability

The authors also would like to acknowledge University of Denver for creating and maintaining a public repository with a comprehensive list of the FEAT datasets and associated documentation.

## HIGHLIGHTS

- Current vehicular ammonia emissions in US urban inventories are underestimated.
- New NH_3_ emission rates based on roadside measurements of cars and trucks across US.
- Onroad NH_3_ inventory increases roughly by a factor of 2 in urban areas.
- Updated NH_3_ emissions reduce model bias in modeled NH_3_ concentrations.
- Modeled PM_2.5_ increases particularly in winter in the northeast region.
