# Supplementary material for: Sensitivity of air quality to vehicle ammonia emissions in the United States
Source: Atmos Environ (1994). Author manuscript; Available in PMC 2024 Jun 15. (PMC11151733; doi:10.1016/j.atmosenv.2024.120484)
Supplement: Toro_etal_NH3mobile_2024_SI [file NIHMS1983631-supplement-Toro_etal_NH3mobile_2024_SI.docx]

**Supporting Information**

**Sensitivity of Air Quality to Vehicle Ammonia Emissions in the United States**

Claudia Toro^1*^, Darrell Sonntag^2^, Jesse Bash^3^, Guy Burke^4^, Benjamin Murphy^3^, Karl M. Seltzer^5^, Heather Simon^5^, Mark Shephard^6^, Karen E. Cady-Pereira^7^

1. US Environmental Protection Agency, Office of Transportation and Air Quality, Ann Arbor, MI
2. Department of Civil and Construction Engineering, Brigham Young University, Provo, UT
3. US Environmental Protection Agency, Office of Research and Development, RTP, NC
4. US Environmental Protection Agency, Region 2, New York, NY
5. US Environmental Protection Agency, Office of Air Quality Planning and Standards, RTP, NC
6. Environment and Climate Change Canada, Toronto, Canada
7. Atmospheric and Environmental Research, Lexington, MA

*Corresponding author

**S1. Datasets**

**S1.1. Light-duty Gasoline Vehicles**

The strength of the FEAT remote sensing device (RSD) for emissions inventory development is its ability to measure emissions from thousands of in-use vehicles, including high emitting vehicles that contribute disproportionately to the emissions inventory^1^. FEAT measures vehicle tailpipe emission concentrations relative to carbon dioxide (CO_2_) from the side of the roadway, typically a single-lane freeway on-ramp, with an infrared and ultraviolet light-source on one side of the roadway and detectors on the other. CO_2_, carbon monoxide, and hydrocarbons emissions are measured using a nondispersive infrared detector and NH_3_ emissions are measured with a dispersive ultraviolet spectrometer. Using the measured carbon emissions and the carbon content of the fuel, the NH_3_ emission rates are estimated relative to fuel consumption in units of grams of pollutant per kilogram of fuel-burned (g/kg-fuel).

The emissions data collected by FEAT emission measurement campaigns is publicly available and contains over 335,000 light-duty gasoline vehicle-specific NH_3_ observations made in seven locations across the United States conducted from 2005 to 2020. Figure S- 1 shows the number of measurements by location and calendar year. Since this analysis was conducted, additional data from campaigns conducted in 2020 and 2021 have been posted, which have not been incorporated into the analysis.


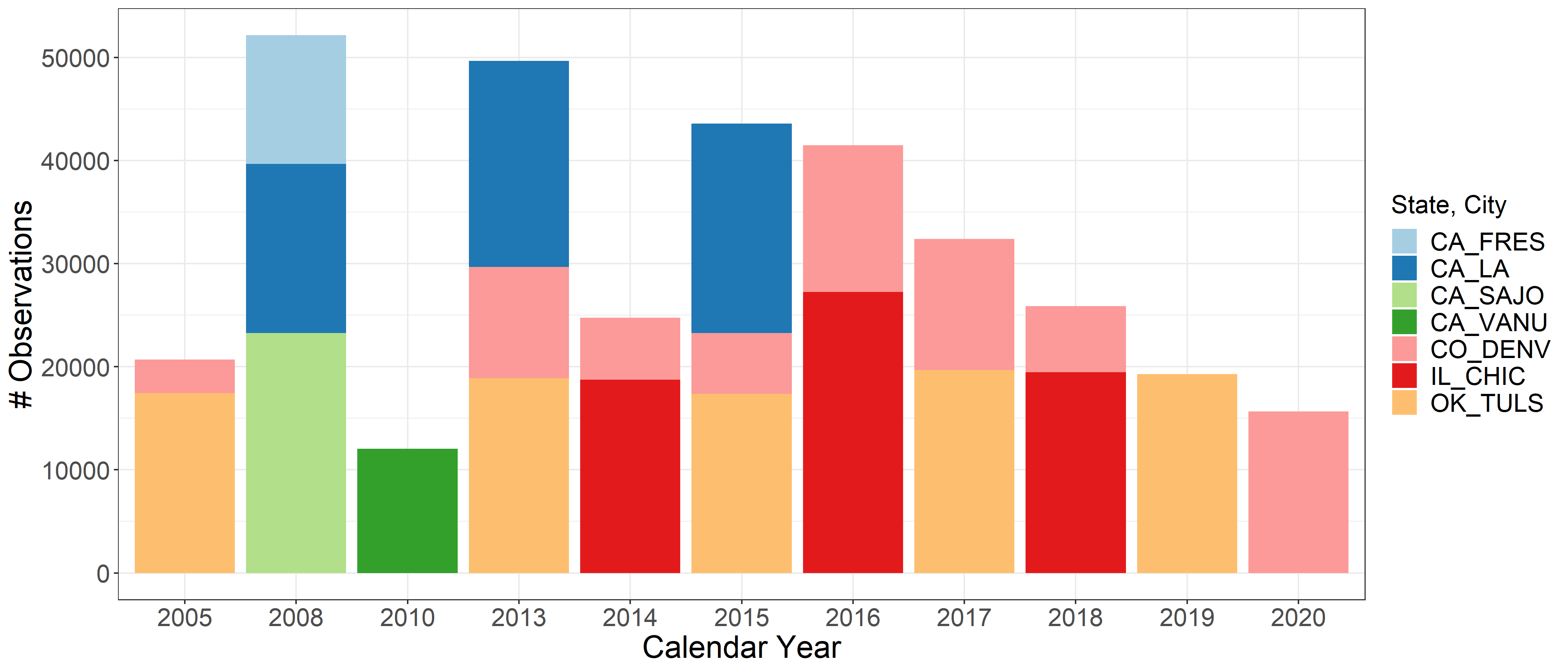


Figure S- 1. Number of Vehicle Ammonia Measurements by Location and Calendar Year. CA_FRES: Fresno, California; CA_LA: Los Angeles, California; CA_SAJO: San Jose, California; CA_VANU: Van Nuys Tunnel, Los Angeles, California; CO_DENV: Denver, Colorado; IL_CHIC: Chicago, Illinois; OK_TULS: Tulsa, Oklahoma.

Table S- 1. Location, calendar year of University of Denver RSD sampling campaign data analyzed, and fleet average as reported in the literature.

| **Location** | **Year** | **NH_3_ fuel-based emission rate (g/kg)** |
| --- | --- | --- |
| Fresno, California^2^ | 2008 | 0.5 |
| West Los Angeles/La Brea Blvd, California^3^ | 2008, 2013, 2015 | 0.79, 0.58, 0.7 |
| San Jose, California^2^ | 2008 | 0.5 |
| Van Nuys, California^4^ | 2010 | 0.59 |
| Denver, Colorado^5,6^ | 2005, 2013, 2015, 2017, 2020 | 0.47, 0.44, 0.42, 0.37, 0.34 |
| Chicago, Illinois^7^ | 2014, 2016, 2018 | 0.71, 0.64, 0.61 |
| Tulsa, Oklahoma^8^ | 2005, 2013, 2015, 2017, 2019 | 0.5, 0.43, 0.37, 0.37, 0.34 |

**S1.2 Heavy-duty Diesel Vehicles**

We used the fuel-based NH_3_ emission rates from heavy-duty diesel vehicles reported by Preble et al.^9^ at the Caldecott Tunnel near Oakland, CA. The heavy-duty vehicles were sampled as they entered the tunnel at a 4% grade traveling between 30 and 75 mph. They measured elevated CO_2_ concentrations with a nondispersive infrared detector and NH_3_ concentrations using a cavity ring-down spectrometer from the exhaust plumes, from which they estimated NH_3_ fuel-based emission rates using the carbon content of diesel fuel.

Table S-2 Fuel-based NH_3_ emission rates (± 95% Confidence Intervals) from heavy-duty vehicles by aftertreatment and engine model year measured at the Caldecott Tunnel by Preble et al. (2019) ^9^

| **Aftertreatment** | **Engine Model Year** | **NH_3_ (g/kg) fuel-based emission rate** | **Number of vehicles** | **Model year ranges used in MOVES** |
| --- | --- | --- | --- | --- |
| DPF + SCR | 2010-2018 | 0.18 + 0.07 | 547 | 2010-2060 |
| DPF | 2007-2009 | 0.00 + 0.01 | 181 | 2007-2009 |
| Retrofit DPF | 1994-2006 | 0.01 + 0.01 | 114 | Not used |
| No DPF | 2004-2006 | 0.00 + 0.01 | 24 | 2004-2006 |
| No DPF | 1965-2003 | 0.02 + 0.02 | 62 | 1960-2003 |

The fleet average NH_3_ emission rates in Preble et al. compared well to three other studies of heavy-duty vehicles conducted in the US since 2015 as shown in Table S-3 and further discussed in the technical documentation for MOVES4^10^.

**Table S-3 Fleet-average fuel-based NH_3_ emission rates (± 95% Confidence Intervals) from heavy-duty vehicles reported from recent studies.**

| Study | Study Year | Location | Number of vehicles | Heavy-duty vehicle fleet average NH_3_ emission rate (g/kg-fuel) |
| --- | --- | --- | --- | --- |
| Preble et al. (2019) | 2018 | Caldecott Tunnel near Oakland, CA | 1,186 | 0.10 + 0.03 |
| Haugen et al. (2018)^11^ | 2017 | Peralta Weigh Station near Anaheim, CA | 1,844 (HDV)  471 (MDV)  1,408 (high)  907 (low) | 0.09 + 0.02 (HDV)  0.06 + 0.05 (MDV  0.08 + 0.02 (high)  0.06 + 0.05 (low) |
| Bishop et al. (2022)^12^ | 2020 | Perry Weight Station Salt Lake City, UT | 1,591 (HDV)  103 (MDV)  1,053 (high HDV)  538 (low HDV) | 0.08 + 0.06 (HDV)  0.22 + 0.23 (MDV)  0.009 + 0.009 (high HDV)  0.23 + 0.02 (low HDV) |
| Wang et al. (2019)^13^ | 2015 | Fort McHenry Tunnel, Baltimore, Maryland | NA | 0.10 + 0.07 (winter)  0.03 + 0.08 (summer) |

**S1.3 Conversion of fuel-based emission rates (g/kg) to mass rates (g/hr)**

The general approach for transforming fuel-based emission rates from the field campaigns to units of g/hr used as input to the model involves using MOVES fuel consumption rates appropriate for the regulatory class (vehicle type), and model year as shown in Equation S-1 below:

$$\overline{ER}_{regclass,MY, op}\left[ \frac{g}{hr} \right]={Fuel rate}_{regclass,MY,op}\left[ \frac{kgfuel}{hr} \right]\times\overline{Fuel ER}_{regclass,MY}\left[ \frac{g}{kgfuel} \right]$$

We note that in the case of LD vehicles, the dataset contained enough depth to estimate emission rates by age groups. In addition, for smaller fleets like LD diesel, HD gasoline and other vehicles using alternative fuels (E-85, CNG) we made assumptions based on data availability. Further details are discussed in the technical documentation for MOVES4.^10,14^

**S2. Description of National MOVES Runs**

For the national MOVES3 modeling presented in Figure 1, we conducted the MOVES runs using the nation as the geographic domain, with national default inputs, including temperature, relative humidity, vehicle age distributions, vehicle fleet mix, average speed distributions, road type distributions, and hoteling activity (overnight idling activity by long combination trucks^10^).

When the nation is selected as the geographic domain, MOVES simulates vehicle emissions assuming that the vehicle age distribution, vehicle fleet mix, road type distribution, fuel properties, temperature and relative humidity are uniform across the lower 48 states.

For the EPA NEI and the emissions modeling platform, MOVES emissions are estimated using the SMOKE-MOVES process.^15,16^ In this methodology, separate MOVES runs are conducted for individual or groups of counties with local inputs, including vehicle age distributions, vehicle fleet mixes, and local fuel properties. In addition, the SMOKE-MOVES process estimates emissions using the local speed distribution, vehicle miles traveled on different road types, and meteorology. The SMOKE-MOVES process enables calculation of more resolved emissions that account for the varying MOVES inputs across the country.

The MOVES3 runs conducted for this sensitivity case are suited for estimating scaling factors for conducting this study. For future emission inventory development, we recommend using MOVES4 (or later versions) coupled with local vehicle activity information in the SMOKE-MOVES process.

**S3. Scaling Factors (SF) Derived from MOVES Simulations**

Table S-3 contains the NH_3_ SF calculated by applying Equation 1 by vehicle groups and calendar years. For applying these scaling factors to the air quality simulations, we calculated SF by two sub-groups: diesel total and non-diesel total. Diesel total SF are calculated using the emissions from both HD and LD diesel. Because HD diesel emissions contribute most emissions from on-road diesel, the diesel total SF is very similar to the HD diesel SF. The non-diesel total ratios are calculated using the emissions from HD CNG, HD gasoline, LD E85 and light-duty gasoline. Because LD gasoline contributes to most of the NH_3_ emissions from the non-diesel total category, the SF for LD gasoline is very similar to the non-diesel total. We note that since NH_3_ is only produced during the running emission process, using a single scaling factor for each sector is appropriate. For other pollutants (e.g., NOx) emitted during more than one emission process, it might be more adequate to consider scaling emission processes individually.

Table S- 4 NH_3_ emissions scaling factors used in this sensitivity study estimated by vehicle group and calendar year.

|  | **Calendar Year** | | | |
| --- | --- | --- | --- | --- |
| **Vehicle_group** | **2010** | **2017** | **2024** | **2035** |
| HD diesel | 0.27 | 1.53 | 2.02 | 2.10 |
| LD diesel | 0.48 | 1.93 | 2.00 | 1.61 |
| Diesel total | 0.27 | 1.53 | 2.02 | 2.08 |
| HD CNG | 5.36 | 4.07 | 3.07 | 2.65 |
| HD gasoline | 1.89 | 2.15 | 1.96 | 1.68 |
| LD E85 | 2.57 | 2.55 | 1.93 | 1.44 |
| LD gasoline | 1.90 | 2.20 | 1.79 | 1.45 |
| Non-diesel total | 1.90 | 2.20 | 1.80 | 1.46 |
| **Total** | **1.77** | **2.13** | **1.83** | **1.56** |

For the air quality simulation conducted for CY 2017 discussed in the results section, we adjusted the emissions using a scaling factor of 1.54 for on-road diesel and 2.08 for non-diesel emissions. The values used are less than 6% different than the scaling factors presented in Table S-3 for on-road diesel (1.53) and on-road non-diesel (2.20). The scaling factors used for the air quality simulation (1.54 and 2.08) were developed using a first draft of the updated NH_3_ emission rates. We subsequently made minor changes to the final MOVES4 LD gasoline and HD diesel emission rates to correct for slight errors in the first draft and extend the range of the emission rates to cover a larger range of model years. In addition, the final MOVES4 emission rates include updates to the LD diesel, HD CNG, HD gasoline, and LD E85 vehicle groups, which were not included in the first draft emission rates. We did not update the air quality modeling simulations with the updated scaling factors because we expected negligible changes to the air quality modeling results due to the change. However, we report the corrected scaling factors because they will be more consistent with the NH_3_ emission rates that were incorporated in MOVES4. We note that the MOVES model is an ongoing project, and future versions of MOVES may update the NH_3_ emissions further based on additional studies and analysis.

**S4. Detailed Comparison of Emissions from MOVES Simulations**

The annual NH_3_ emissions estimated using the default MOVES3 emission rates (MOVES3) and the updated NH_3_ emission rates (Sensitivity) for calendar years 2010, 2017, 2024, and 2035 are shown by vehicle type and model year in Figure S-2. The emissions increased notably for the newest model years for the sensitivity case for both LD gasoline and HD diesel vehicles. The following figures evaluate the emission differences, the emission rates and activity for these two vehicle groups that contribute most of the on-road NH_3_ emissions: LD gasoline and HD diesel.


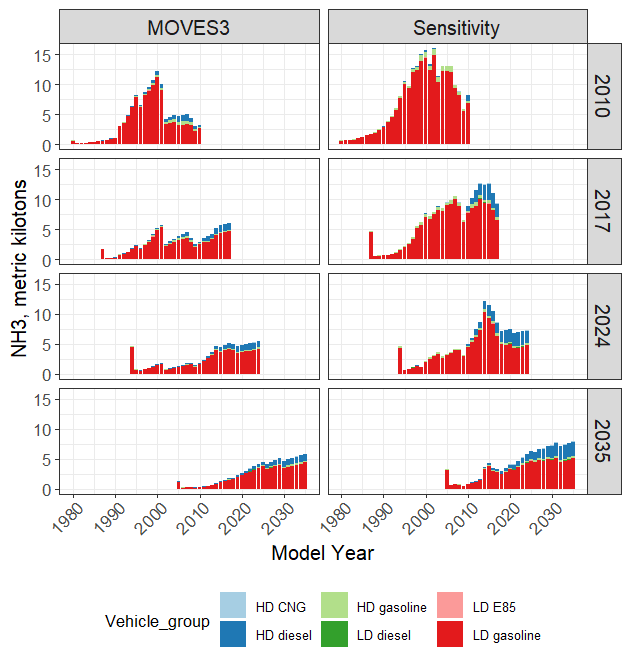


Figure S- 2 Annual National On-road Vehicle NH_3_ Emissions estimated from MOVES3 and the Sensitivity Case by Calendar Year and Vehicle Group. The Vehicle groups are a combination of vehicle type and fuel type. HD = heavy-duty; LD = light-duty. CNG = compressed natural gas; E85 = ethanol-gasoline blend with ~85% ethanol.

Figure S- 3 displays the differences in the national running exhaust NH_3_ emissions (metric kilotons) estimated using MOVES3 and the sensitivity case with the updated NH_3_ emission rates. The differences are calculated by subtracting the running exhaust NH_3_ emissions from the sensitivity case from the NH_3_ emissions from the MOVES3 scenario. Except for the pre-2010 model year diesel emissions, the sensitivity case NH_3_ emissions are larger than the MOVES3 emissions.


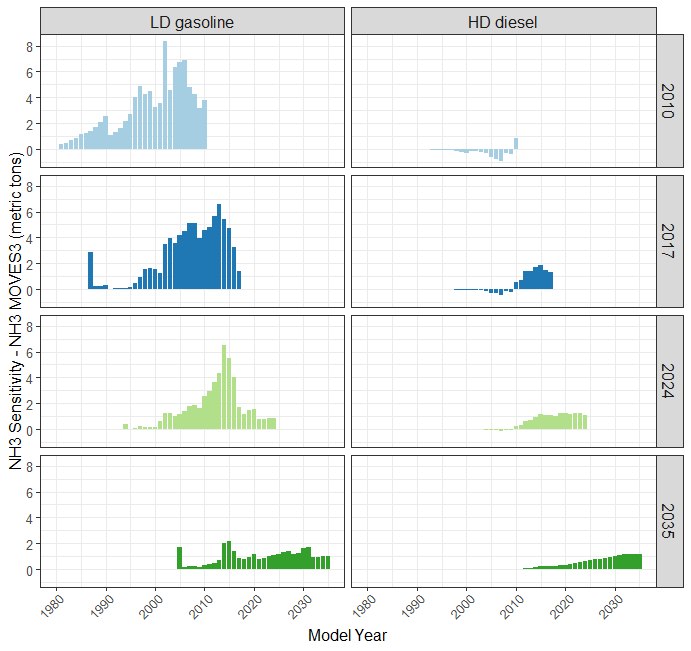


Figure S- 3. Difference in the National NH_3_ running exhaust emission (metric kilotons) by model year for gasoline and diesel vehicles calculated by MOVES3 for the MOVES3 and the Sensitivity Case

The largest increase in NH_3_ emissions in the sensitivity case occur for the gasoline vehicles between model year 2000 and 2016 for calendar years 2010, 2017, and 2024. We evaluated the emission rates (g/km) and the activity (kilometers driven) by model year and calendar year for these two groups to better understand the emission differences.

Figure S- 4 displays the average NH_3_ exhaust emission rates for light-duty (LD) gasoline and heavy-duty (HD) diesel vehicles by model year. The average emission rates were calculated from the MOVES simulations for the MOVES3 baseline and updated NH_3_ rates (Sensitivity) scenarios. For gasoline vehicles, the updated NH_3_ emission rates are larger than the MOVES3 rates for all model years of the three calendar years evaluated. The largest increases in the updated gasoline emission rates between the two scenarios are for the 1980 to 1990 model years, and the model year 2002-2016 model years. For diesel vehicles, the updated NH_3_ exhaust emission rates are substantially larger than the MOVES3 rates for all model years starting in 2010.


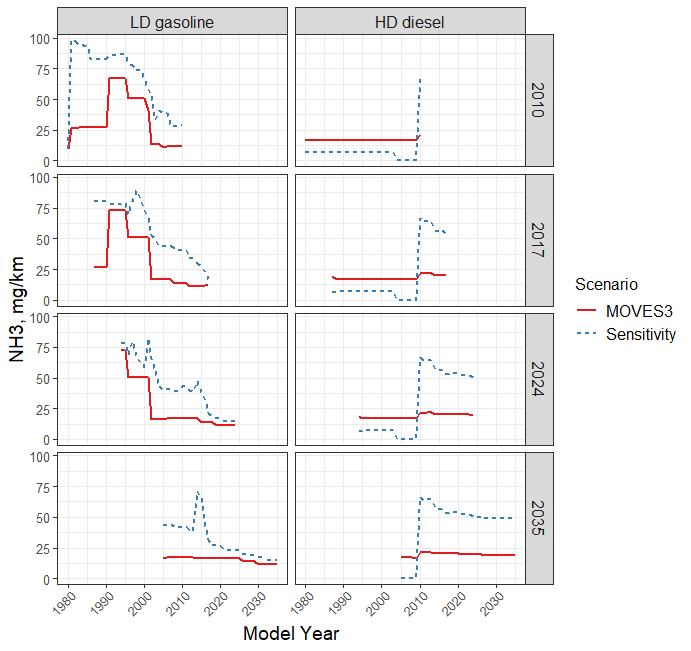


Figure S- 4. Average NH_3_ running exhaust emission rates (mg/km) by model year for gasoline and diesel vehicles calculated by MOVES3 for the default MOVES3 and the Sensitivity Case

Figure S- 5 displays the vehicle kilometers traveled for the LD gasoline and HD diesel vehicles by model year for the MOVES3 and sensitivity scenarios. The vehicle activity, including population and vehicle kilometers traveled, are unchanged between the two scenarios. As shown, the LD gasoline vehicles dominate the number of vehicles traveled. In general, the most recent model years contribute the most vehicle kilometers traveled, with exceptions for the ~ 2010 model years due to the ‘great recession.’


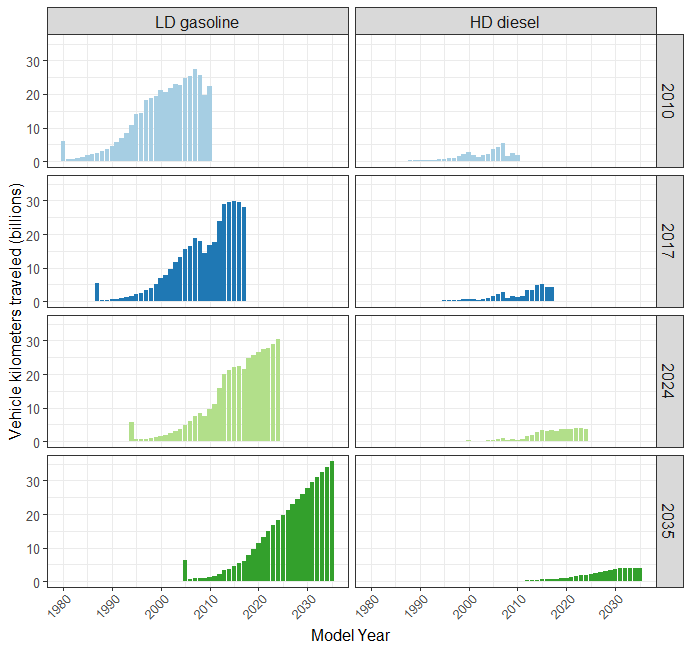


Figure S- 5. National vehicle kilometers traveled (billions of kilometers) by model year for gasoline and diesel vehicles for both the MOVES3 and the Sensitivity Case

The large increase in NH_3_ emissions in the sensitivity case occur for the gasoline vehicles between model year 2000 and 2016 shown in Figure S-3 are due to the increase in emission rates (Figure S-4) coupled with these vehicles having a large share of the total kilometers traveled (Figure S- 5). In 2035, the model year 2000 and 2016 gasoline vehicles no longer contribute substantially to the kilometers driven of the in-use fleet, and thus these model year vehicles no longer contribute substantially to the total NH_3_ emissions.

Starting in 2010 model year vehicles, heavy-duty diesel vehicles are estimated to have higher NH_3_ emission rates (g/km) than light-duty vehicles. However, as shown in Figure S- 5 diesel vehicles travel much less kilometers than gasoline vehicles, leading to a lower contribution of total running NH_3_ emissions from diesel vehicles, especially in historic years. As time progresses, diesel vehicles contribute to an increasingly larger share of NH_3_ emissions (Figure S- 2), which is especially evident in 2035, where nearly all the diesel vehicle kilometers driven are projected to be by vehicles newer than model year 2010 (Figure S- 5).

**S5. Evaluation against CrIS satellite and ground-based network observation**

| 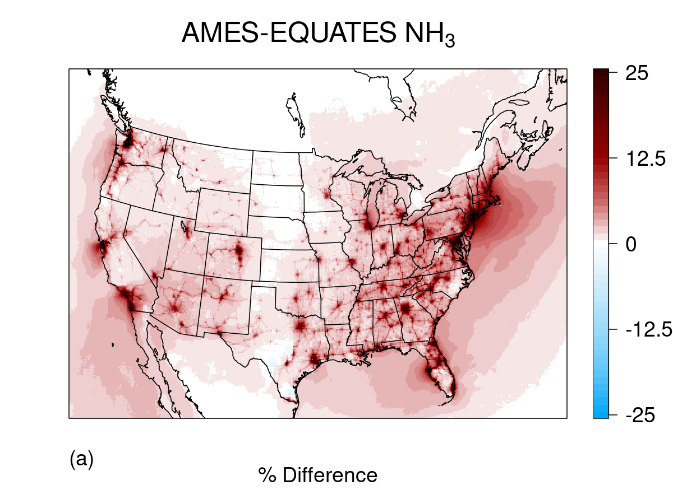 | 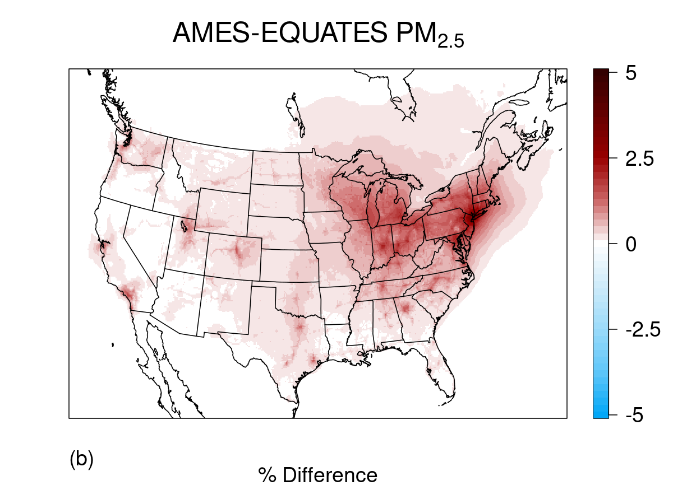 |
| --- | --- |
| 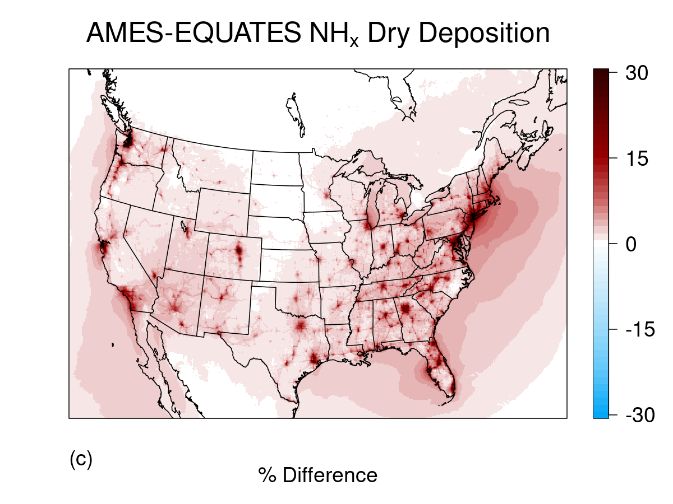 | 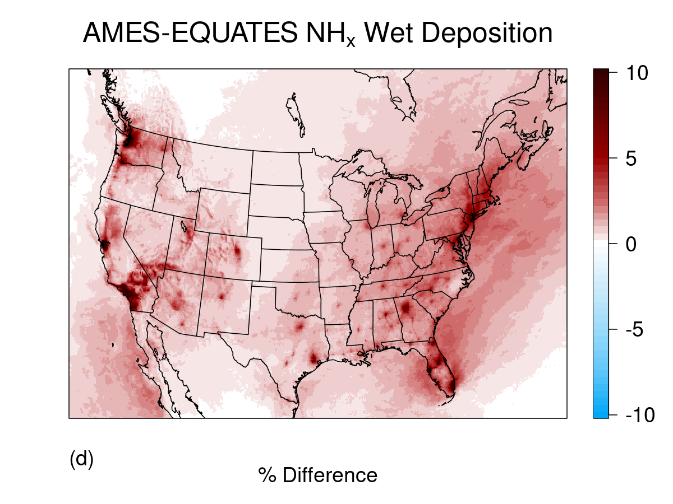 |

**Figure S- 6**. **Annual model percent differences (AMES – EQUATES)/EQUATES 100% in surface layer NH3 concentrations (a), PM2.5 (b), NHx, NH3 + Aerosol NH4+, dry deposition (c), NHx wet deposition (d)**

| 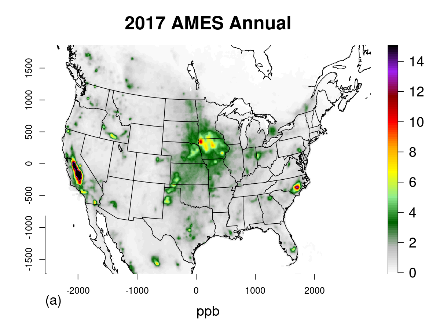 | 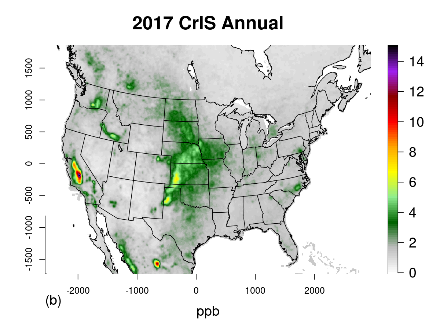 | 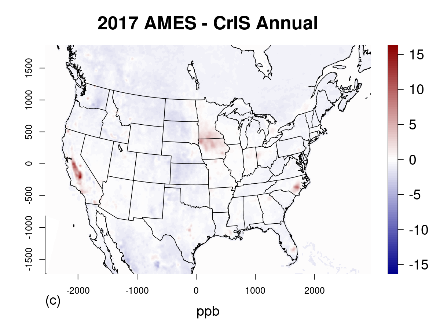 |
| --- | --- | --- |

Figure S- 7. Annual mean 2017 CrIS NH_3_ (ppb_v_) surface layer retrievals mapped onto the EQUATES model domain grid (a), annual mean 2017 CMAQ v5.3.2 AMES case (b), 2017 annual mean CMAQ – CrIS NH_3_ surface layer concentrations differences. The CMAQ modeled results were paired in space and time with the CrIS satellite retrieval (approximately 13:00 local time when conditions permitted a successful retrieval)

| 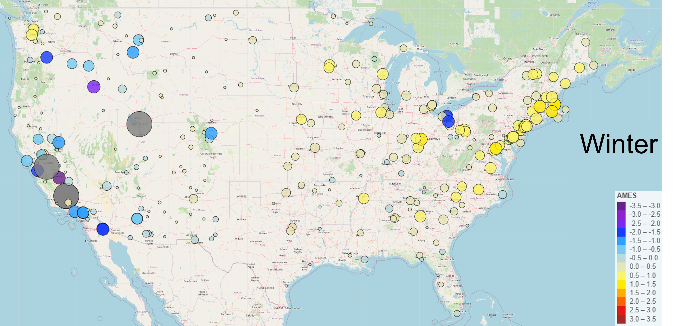 | 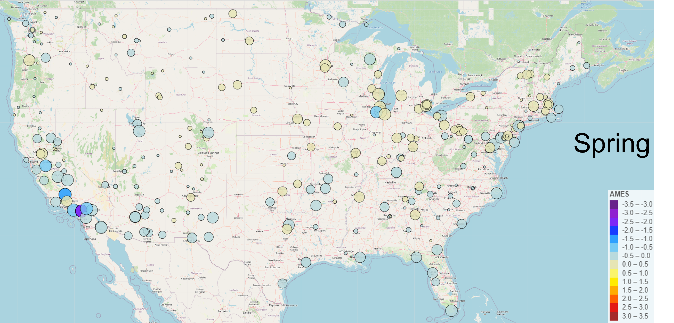 |
| --- | --- |
| 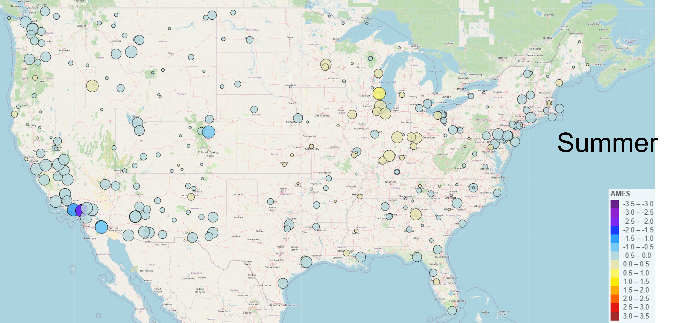 | 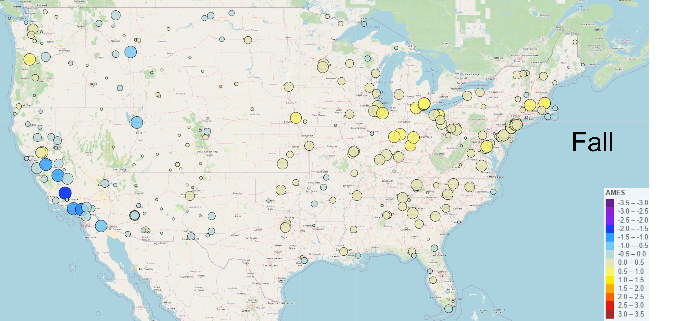 |

**Figure S-8. Seasonal PM2.5 Nitrate bias (AMES-AQS Observations) for December, January and February (a), March, April, and May (b), June, July, and August (c), and September, October, and November (d). Positive values in yellows and reds indicate model overestimates and negative values in blues and purples indicate model underestimates in AQS observations in µg m^-3^. The size of the point is proportional to the model bias at the AQS site and grey shaded circles represent biases that exceed the range on the legend (All values are model underestimates).**

| 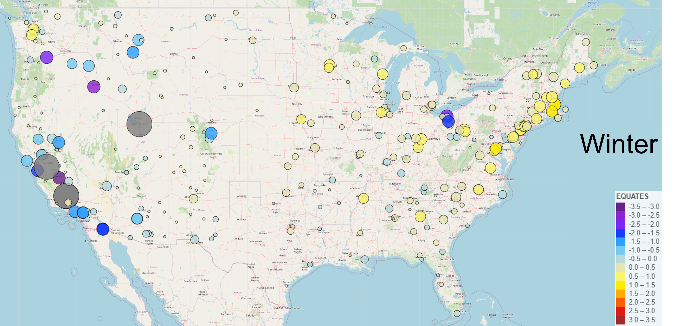 | 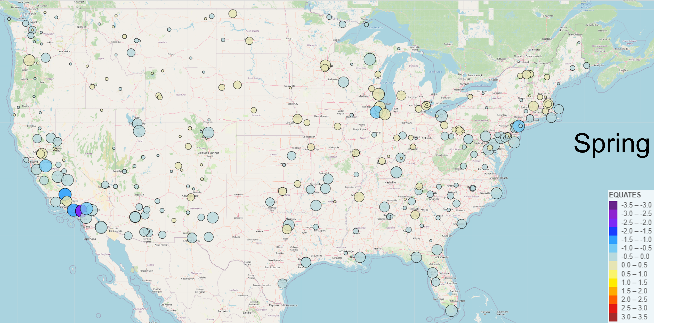 |
| --- | --- |
| 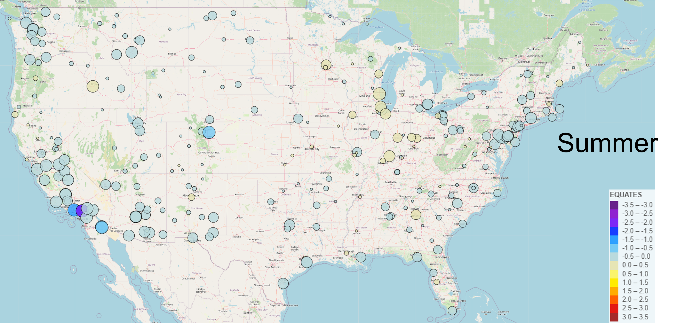 | 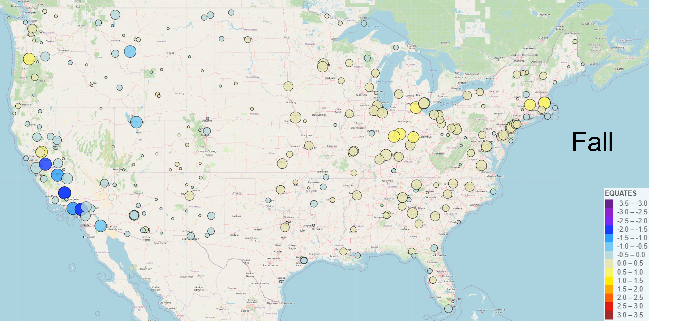 |

**Figure S-9. Seasonal PM2.5 Nitrate bias (EQUATES-AQS Observations) for December, January and February (a), March, April, and May (b), June, July, and August (c), and September, October, and November (d). Positive values in yellows and reds indicate model overestimates and negative values in blues and purples indicate model underestimates in AQS observations in µg m^-3^. The size of the point is proportional to the model bias at the AQS site and grey shaded circles represent biases that exceed the range on the legend (All values are model underestimates).**

| 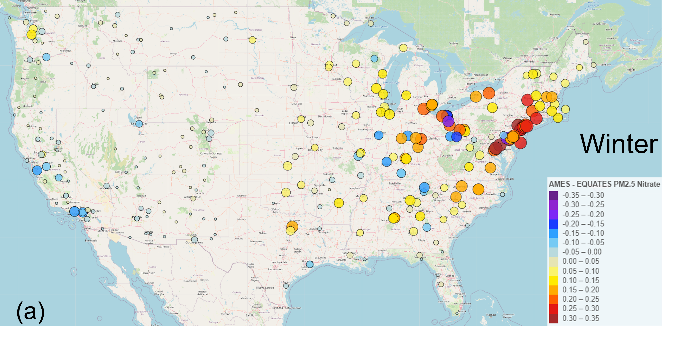 | 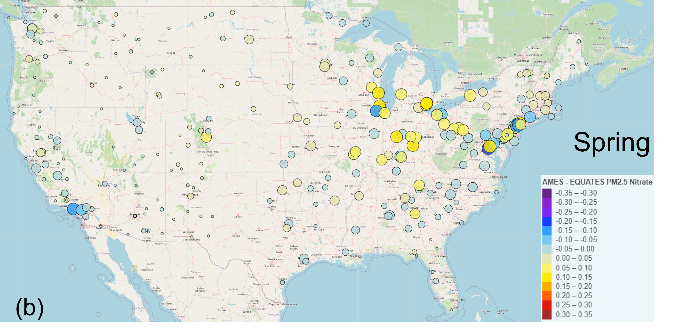 |
| --- | --- |
| 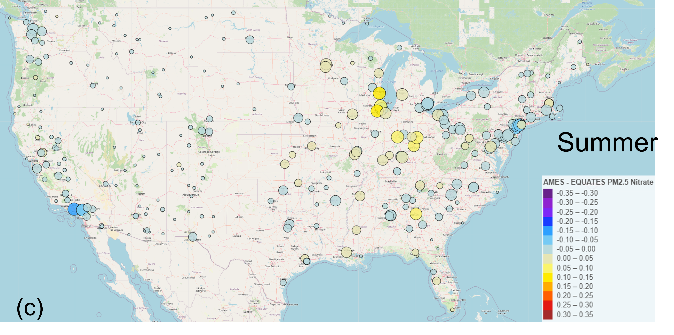 | 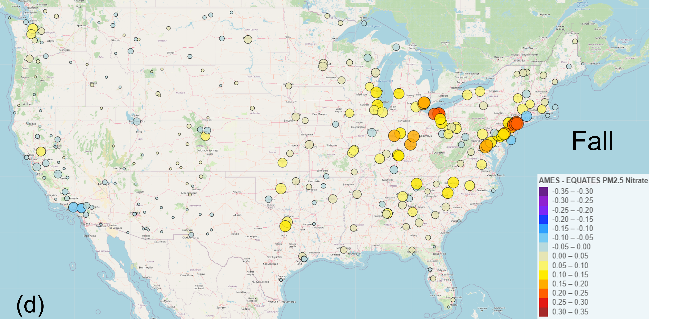 |

**Figure S-10 Season change in absolute PM2.5 Nitrate bias (AMES-EQUATES) for December, January and February (a), March, April, and May (b), June, July, and August (c), and September, October, and November (d). Positive values in yellows and reds indicate an increase and negative values in blues and purples indicate a decrease in the AMES sensitivity model bias in µg m^-3^. The size of the point is proportional to the model bias at the AQS site.**

| 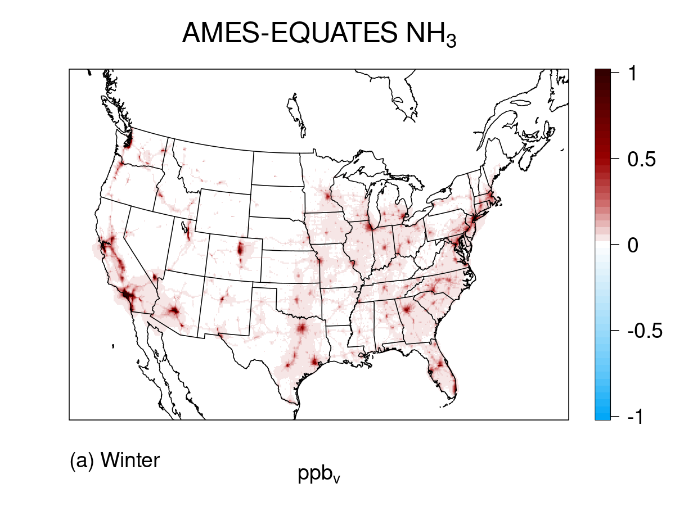 | 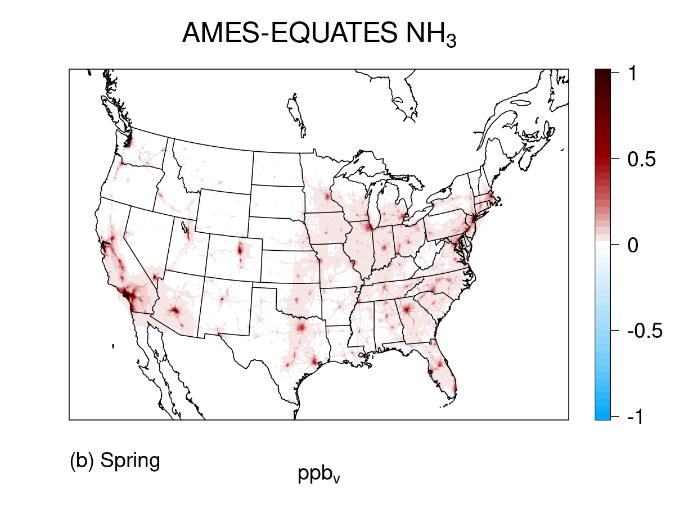 |
| --- | --- |
| 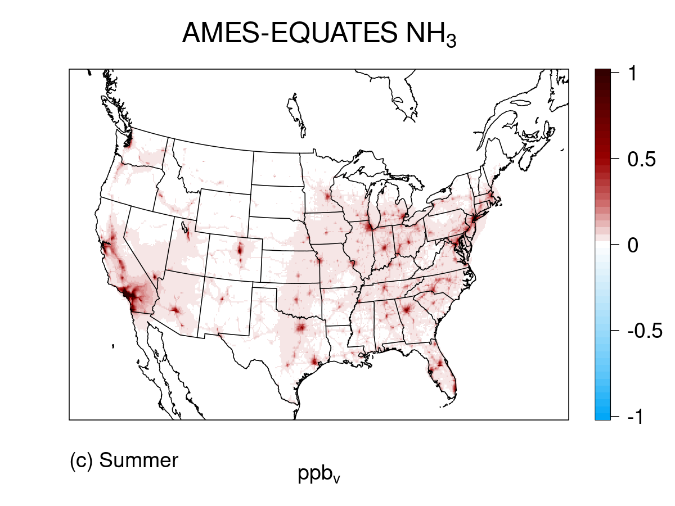 | 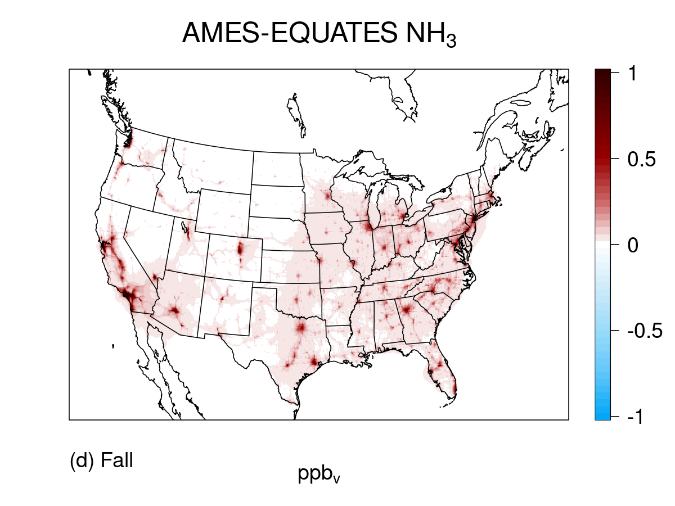 |

**Figure S- 11**. **Seasonal model differences (AMES – EQUATES) in surface layer NH3 concentrations ppb_v_ December, January, and February (a), March, April, and May (b), June, July, and August (c), September, October, and December (d)**

**References**

(1) *Fuel Efficiency Automobile Test Data Repository | University of Denver Research | Digital Commons @ DU*. https://digitalcommons.du.edu/feat/ (accessed 2022-04-27).

(2) Stedman, D.; Bishop, G.; Peddle, A. *On-Road Motor Vehicle Emissions Including NH3, SO2 and NO2*; California Air Resources Board: Sacramento, CA, 2009. https://digitalcommons.du.edu/feat_publications/64.

(3) Bishop, G.; Stedman, D. *Measuring Real-World Emissions from the On-Road Passenger Fleet*; 2016. https://ww2.arb.ca.gov/sites/default/files/classic/research/apr/past/17rd015.pdf.

(4) Bishop, G. A.; Schuchmann, B. G.; Stedman, D. H.; Lawson, D. R. Multispecies Remote Sensing Measurements of Vehicle Emissions on Sherman Way in Van Nuys, California. *J. Air Waste Manag. Assoc.* **2012**, *62* (10), 1127–1133. https://doi.org/10.1080/10962247.2012.699015.

(5) Bishop, G. A. *On-Road Remote Sensing of Automobile Emissions in the Denver Area: Winter 2020*; Coordinating Research Council, 2020. http://crcao.org/wp-content/uploads/2020/06/University-of-Denver-CRC-123-Denver-2019-Final-report-v1.pdf.

(6) Bishop, G. A.; Stedman, D. H. Reactive Nitrogen Species Emission Trends in Three Light-/Medium-Duty United States Fleets. *Environ. Sci. Technol.* **2015**, *49* (18), 11234–11240. https://doi.org/10.1021/acs.est.5b02392.

(7) Bishop, G. A. *On-Road Remote Sensing of Automobile Emissions in the Chicago Area: Fall 2018*; CRC Report No. E-123; Coordinating Research Council, Inc., 2019. https://crcao.org/wp-content/uploads/2019/10/E-123-Chicago-2018-Final-Report-v1_06.26.19.pdf.

(8) Bishop, G. *On-Road Remote Sensing of Automobile Emissions in the Tulsa Area: Fall 2019*; CRC E-123; Coordinating Research Council, Inc. https://crcao.org/wp-content/uploads/2020/08/University-of-Denver-CRC-123-Tulsa-2019-Final-report.pdf.

(9) Preble, C. V.; Harley, R. A.; Kirchstetter, T. W. Control Technology-Driven Changes to In-Use Heavy-Duty Diesel Truck Emissions of Nitrogenous Species and Related Environmental Impacts. *Environ. Sci. Technol.* **2019**, *53* (24), 14568–14576. https://doi.org/10.1021/acs.est.9b04763.

(10) US Environmental Protection Agency. *Exhaust Emission Rates of Heavy-Duty Onroad Vehicles in MOVES4*; EPA-420-R-23-027; 2023. https://www.epa.gov/moves/moves-onroad-technical-reports.

(11) Haugen, M. J.; Bishop, G. A.; Thiruvengadam, A.; Carder, D. K. Evaluation of Heavy- and Medium-Duty On-Road Vehicle Emissions in California’s South Coast Air Basin. *Environ. Sci. Technol.* **2018**, *52* (22), 13298–13305. https://doi.org/10.1021/acs.est.8b03994.

(12) Bishop, G. A.; Haugen, M. J.; McDonald, B. C.; Boies, A. M. Utah Wintertime Measurements of Heavy-Duty Vehicle Nitrogen Oxide Emission Factors. *Environ. Sci. Technol.* **2022**, *56* (3), 1885–1893. https://doi.org/10.1021/acs.est.1c06428.

(13) Wang, X. Real-World Vehicle Emissions Characterization for the Shing Mun Tunnel in Hong Kong and Fort McHenry Tunnel in the United States. *Res. Rep.* **2019**.

(14) US Environmental Protection Agency. *Exhaust Emission Rates for Light-Duty Onroad Vehicles in MOVES4*; EPA-420-R-23-028; 2023. https://www.epa.gov/moves/moves-onroad-technical-reports.

(15) US EPA. *2017 National Emissions Inventory: January 2021 Updated Release, Technical Support Document*; EPA-454/R-21-001; 2021. https://www.epa.gov/sites/default/files/2021-02/documents/nei2017_tsd_full_jan2021.pdf.

(16) Eyth, A.; Vukovich, J.; Farkas, C.; Strum, M. *Technical Support Document (TSD) Preparation of Emissions Inventories for 2016v1 North American Emissions Modeling Platform*; 2020. https://www.epa.gov/sites/default/files/2020-10/documents/preparation_of_emissions_inventories_for_2016v1_north_american_emissions_modeling_platform_tsd.pdf.
